# Cortico-striatal differences in the epigenome in attention-deficit/ hyperactivity disorder

**DOI:** 10.1038/s41398-024-02896-x

**Published:** 2024-04-11

**Authors:** Gauri G. Shastri, Gustavo Sudre, Kwangmi Ahn, Benjamin Jung, Bhaskar Kolachana, Pavan K. Auluck, Laura Elnitski, Stefano Marenco, Philip Shaw

**Affiliations:** 1 Social and Behavioral Research Branch, National Human Genome Research Institute, NIH, Bethesda, MD 20892 USA; 2 Human Brain Collection Core, National Institute of Mental Health, NIH, Bethesda, MD 20892 USA; 3 Translational and Functional Genomics Branch, National Human Genome Research Institute, NIH, Bethesda, MD 20892 USA

**Keywords:** Epigenetics and behaviour, ADHD

## Abstract

While epigenetic modifications have been implicated in ADHD through studies of peripheral tissue, to date there has been no examination of the epigenome of the brain in the disorder. To address this gap, we mapped the methylome of the caudate nucleus and anterior cingulate cortex in post-mortem tissue from fifty-eight individuals with or without ADHD. While no single probe showed adjusted significance in differential methylation, several differentially methylated regions emerged. These regions implicated genes involved in developmental processes including neurogenesis and the differentiation of oligodendrocytes and glial cells. We demonstrate a significant association between differentially methylated genes in the caudate and genes implicated by GWAS not only in ADHD but also in autistic spectrum, obsessive compulsive and bipolar affective disorders through GWAS. Using transcriptomic data available on the same subjects, we found modest correlations between the methylation and expression of genes. In conclusion, this study of the cortico-striatal methylome points to gene and gene pathways involved in neurodevelopment, consistent with studies of common and rare genetic variation, as well as the post-mortem transcriptome in ADHD.

## Introduction

While there have been marked advances in the understanding of how common and rare genetic variation confers risk for attention-deficit/hyperactivity disorder (ADHD) [1, 2], much less is known about the role of epigenetic changes. There are several reasons to think epigenetic processes, such as the methylation of DNA, may play a role [3]. Firstly, epigenetic regulation is associated with key processes in the human central nervous system including neurogenesis [4] and fetal brain development [5]. While there are many potential epigenetic mechanisms at play, we focus on the methylation of DNA at the C-5 position of the cytosine ring, the most widely studied epigenetic event in psychiatric genomics. Subtle perturbations in these processes are a potential mechanism for neurodevelopmental conditions that usually have their onset early in life, such as ADHD. In this context, it is notable that childhood ADHD symptoms have been associated with methylomic change in cord blood obtained at birth in neurodevelopmental genes, such as *CREB5* which regulates neurite outgrowth and *SKI* involved in neural tube development [6, 7]. Indeed the prospective association between cord blood methylation and childhood ADHD has been found to be more robust than associations with methylation changes found in peripheral blood [6–11] or saliva [12, 13] acquired during childhood. Such work suggests that early epigenetic modifications to genes in neurodevelopmental pathways may be important for the emergence and course of childhood ADHD.

A major barrier to the interpretation of changes in the peripheral methylome is the absence of studies of the brain’s methylome in ADHD. Epigenetic modifications show considerable tissue specificity and thus changes detected in cord blood, peripheral blood or saliva may not reflect changes that occur in the brain [14]. While rich insights have stemmed from postmortem methylome studies in other neurodevelopmental disorders such as autism spectrum disorders [15] and schizophrenia [16, 17], such work has been lacking in ADHD. Thus, here we aim to give novel insights into potential epigenetic mechanisms in ADHD by providing the first report on the methylome from post-mortem brain tissue on donors with lifetime histories of ADHD. We hypothesized that change may be particularly prominent in genes implicated in neural development.

We examined two brain regions, the anterior cingulate cortex (ACC) and the caudate for four reasons. First, these interconnected regions support many cognitive functions disrupted in ADHD, including inhibitory processing and attention-demanding tasks [18]. Second, both regions have been found in mega and meta-analytic magnetic resonance imaging studies to show structural and functional changes [19–21]. Third, the regions are also enriched for neurotransmitters implicated in ADHD, such as dopamine, the neurotransmitter modulated by psychostimulant medication [22–25], and glutamate, the brain’s major excitatory neurotransmitter [26]. Finally, we have also reported recently on change in gene expression in these same brain regions among the same donors [27]. We found transcriptome-wide differential expression of fourteen genes in the ACC, and one in the caudate, along with an enrichment of gene sets involved in neurodevelopmental processes and in neurotransmission.

Thus, our study has three aims. First, we map ADHD-related changes in the methylome of the post-mortem brain, focusing on corticostriatal regions, determining if these changes implicate processes pertaining to neurodevelopment. Second, we examine if genes implicated by ADHD-related changes in methylation overlap with those implicated by differential transcription, in the same brain regions and the same subjects. Finally, we assess whether the genes implicated by differential methylation overlap with genes implicated through GWAS of ADHD and other genetically correlated psychiatric disorders, particularly other neurodevelopmental disorders, such as autistic spectrum disorders [1, 28–30].

## Methods

### Postmortem brain tissue selection and preparation

The final analyses in the study used postmortem brain tissue from 58 donors, of which 24 (10 cases, 14 controls) were from the National Institute of Mental Health Human Brain Collection Core (HBCC) and 34 were from the Neurobiobank (5 cases, 5 controls from Brain Tissue Donation Program at the University of Pittsburgh, and 10 cases, 14 controls from University of Maryland Brain and Tissue Bank). ADHD diagnosis was determined at each study site by interviewing next of kin using DSM criteria as well as review of prior records – see Supplemental Methods. Exclusion criteria included presence of major neurological disorder or schizophrenia. Controls were defined as those with no history of mental illness.

Brain tissue was sectioned as coronal slabs at autopsy and then frozen at –80 °C. Dissections were performed on frozen tissue held on dry ice in small batches, with each sample on dry ice for about 30 min. Dissections targeted the dorsal anterior cingulate cortex (ACC), above the genu of the corpus callosum, and the head of the caudate. Tissue for methylation analyses was available from the ACC of 55 donors and the caudate of 58 donors. DNA was extracted from bulk tissue homogenates, bisulfite conversion used the EZ DNA Methylation kit and the methylation analyses were conducted at the Genomics Core of the NHGRI using the Infinium HumanMethylationEPIC BeadChip (Illumina, San Diego, CA).

### DNA methylation data processing

Methylation data was processed using the Psychiatric Genomics Consortium (PGC), ADHD Working Group pipeline for EWAS. In summary, raw IDAT files were imported in R using the *minfi* package. Samples were excluded based on the following criteria: (1) low overall intensity (median unmethylated or methylated signal <13), sodium bisulfite conversion median <80, (2) detection *p* value > 0.01 in more than 1% of probes, (3) overall methylation call rate < 95%, and (4) reported sex did not match predicted sex generated using the *minfi* package function *getSex()*. Principal components (PCs) calculated across all probes were used to identify outliers, and any samples >2 standard deviations from the mean for both PC1 and PC2 were removed. In addition, the following probes were removed: (1) probes with >2% of samples with detection *p* value > 0.01, (2) probes annotated to the X and Y chromosomes, (3) probes that cross-hybridize, (4) non-CpG site probes, and (5) probes that overlap with common SNPs. Filtered probes were quantile normalized using the CPACOR pipeline [31]. Finally, we calculated a smoking score from the DNA methylation data [32]. After these QC procedures, we retained 811,639 probes for the caudate and 820,051 for the ACC. Two caudate samples were removed based on median intensity plots, and four ACC samples were removed as outliers based on PCA on SNPs identified from the methylation array data. The final postmortem brain EWAS thus included data from 51 ACC and 56 caudate specimens.

### Examination of differentially methylated probes and regions in ADHD in postmortem brain

Epigenome-wide association study (EWAS) analyses were conducted using the *cpg.assoc* function from the *minfi* R package in which we conducted a series of linear regressions using clinical diagnosis of ADHD as the independent variable and each CpG methylation as the dependent variable, an approach which has proved suitable for the analysis of EPIC array data [33]. ACC and caudate were analyzed separately.

We considered a range of variables that have been associated with DNA methylation in prior studies: demographic/clinical features (age at death, gender, comorbidities, substance abuse, mode of death, clinical evidence level), genotypic (the first five ancestry components - C1 through C5), technical covariates (processing batch, brain bank of origin, post-mortem interval, and the first 10 principal components derived from control probes), and biological covariates (estimated proportion of NeuN+ (neuronal) cells [34] and a DNA methylation-based smoking score) for inclusion in the model. To select the variables, we used the approach employed by our group in previous post-mortem studies and others. Specifically, we extracted the principal components of the methylation data and retained the components with eigenvalues above one (R package nFactors, version 2.4.1). The first ten principal components were retained for the ACC, accounting for 46% of variance, and fourteen principal components were retained for the caudate, accounting for 49% of variance. Spearman correlations tested for associations between these principal components and continuous covariates, while a Kruskal-Wallis test was used for categorical covariates. Covariates associated with any principal component at a Bonferroni corrected *p* value < 0.05 were retained in the final model. For the ACC, proportion of neurons, age at death, and one technical component (PC5) were selected. For the caudate, the approach selected the proportion of neurons, age at death, smoking score, two ancestral components (C1 and C3), and two of the technical components (PC3 and PC4). Finally, we also included variables associated with diagnosis at a Bonferroni corrected *p* value < 0.05: for both brain regions, substance abuse was added to the final model.

### Differentially methylated regions

We employed mCSEA, an algorithm designed to find differentially methylated regions (DMRs) that have a small but consistent delta in methylation related to complex phenotypes [35], and investigated which regions of the methylome are differentially related to ADHD. Methylation sites were classified as promoters when column UCSC_RefGene_Group in the data package IlluminaHumanMethylationEPICanno.ilm10b2.hg19 contained terms: TSS1500, TSS200, 5ʹ untranslated region [UTR], or 1stExon; classified as belonging to gene bodies if the same column had the term “Body”, and taken as CGI if the column “Relation_to_Island” was either Island, N_Shore, S_Shore, N_Shelf, or S_Shelf. mCSEA ranks all CpG sites based on the t-statistic assessing the association between methylation and phenotype (using R *limma*) and performs an enrichment analysis on CpG sites in the pre-defined regions using GSEA (implemented in *fgsea*). Regions with CpG sites over-represented in the ordered list of sites emerge as DMRs. The analyses for DMRs were conducted separately for probes in gene bodies, promoter regions, and CGIs. All probes were considered in these analyses, but only regions with 5 or more probes were analyzed. For the ACC, this resulted in 15,155 regions for gene bodies, 19,162 for promoters, and 24,657 for CGIs. For the caudate, this resulted in 15,076 regions for gene bodies, 19,073 for promoters, and 24,626 for CGIs. These DMRs were mapped to genes using the leading edge CpG probes that contribute most to its differential methylation. Specifically, the leading probes are mapped to the nearest gene using the R package IlluminaHumanMethylationEPICanno.ilm10b2.hg19, (within 1500 bp upstream or downstream). The mCSEA tool has been successfully applied to other complex neuropsychiatric phenotypes such post-traumatic stress disorder [36], epilepsy [37], and rare neurogenetic syndromes, such as Cri du Chat [38]. The approach also compares well against other tools for DMR detection in terms of false positive rates [39].

### Gene set enrichment analysis (GSEA)

GSEA were run using *gometh()* in the *missmethyl* package. We included CpG sites that were the leading probes for genes implicated in the mCSEA analyses, retaining those significant at FDR q < 0.05 and running analyses for caudate and ACC separately. *gometh()* is designed specifically to account for sources of bias inherent in methylation analysis, such as the differing number of probes per gene and CpGs sites that are annotated to multiple genes [40]. We then used REVIGO [41] to run a semantic space analysis on the gene sets significant at FDR q < 0.05.

### Relating the post-mortem methylome to the transcriptome

There were 48 samples with both transcriptome and methylome data for the ACC, and 54 for the caudate. These analyses used the *mCSEAIntegrate()* function within mCSEA. First, the DMR is summarized in one value- the average of the methylation difference across the leading edge CpG probes that contribute most to its differential methylation. The DMR is then mapped to a its nearest genes using the R package IlluminaHumanMethylationEPICanno.ilm10b2.hg19 (within 1500 bp upstream or downstream). Next, we calculated the Pearson correlation coefficient between each region’s average methylation and the expression of its nearby genes (within 1500 bp upstream or downstream, again using the same annotation R packages as above). The expected correlation depends on the region type: negative correlation for promoters, as methylation typically suppresses gene expression (Jones & Baylin, 2002); positive correlation for gene bodies, as methylation often increases expression (Aran et al., 2011), and a mix of positive and negative correlations for CpGs within CGIs as these complex regions can contain both promoters and gene bodies.

Details on the transcriptomic analyses are given elsewhere, but in brief the gene expression was conducted using Illumina NovaSeq 6000, 2 × 150 bp following Ribo-Zero GOLD treatment to remove mitochondrial RNA and cytoplasmic rRNA. We used the same analytic approach in the TWAS as in the current MWAS, considering the same variables along with ones unique to RNASeq (RNA-seq batch, and RINe). We report both nominally and FDR adjusted correlations (setting q < 0.05).

### Comparison of differentially methylated genes with genes implicated in psychiatric disorders

Genes implicated in ADHD (at FDR q < 0.05) through the mCSEA DMR analyses were entered into Multi-marker Analysis of GenoMic Annotation (MAGMA) [42]. This analysis tests whether the genes implicated by DMRs were associated with genes for ADHD implicated through GWAS. MAGMA relates GWAS SNPs to genes by their genomic positions, and then uses a SNP-wise sum model to test the SNP association with a gene-level continuous variable reflecting differential methylation. The 2023 Psychiatric Genomics Consortium ADHD GWAS data release was used along with the concatenation of European and African-American samples from 1000Genomes as the reference data to estimate linkage disequilibrium between SNPs [43]. We also used MAGMA to investigate whether differential methylation in ADHD would overlap with genes implicated through GWAS for other psychiatric disorders, specifically autism spectrum disorder [44], major depression [45, 46], bipolar affective disorder [47], schizophrenia [48], Tourette Syndrome [49], obsessive compulsive disorder [50], Alzheimer’s disease, alcohol use disorder, and as a ‘negative’ control, rheumatoid arthritis.

## Results

### Epigenome-wide association study in ADHD

**Fig. 1:**
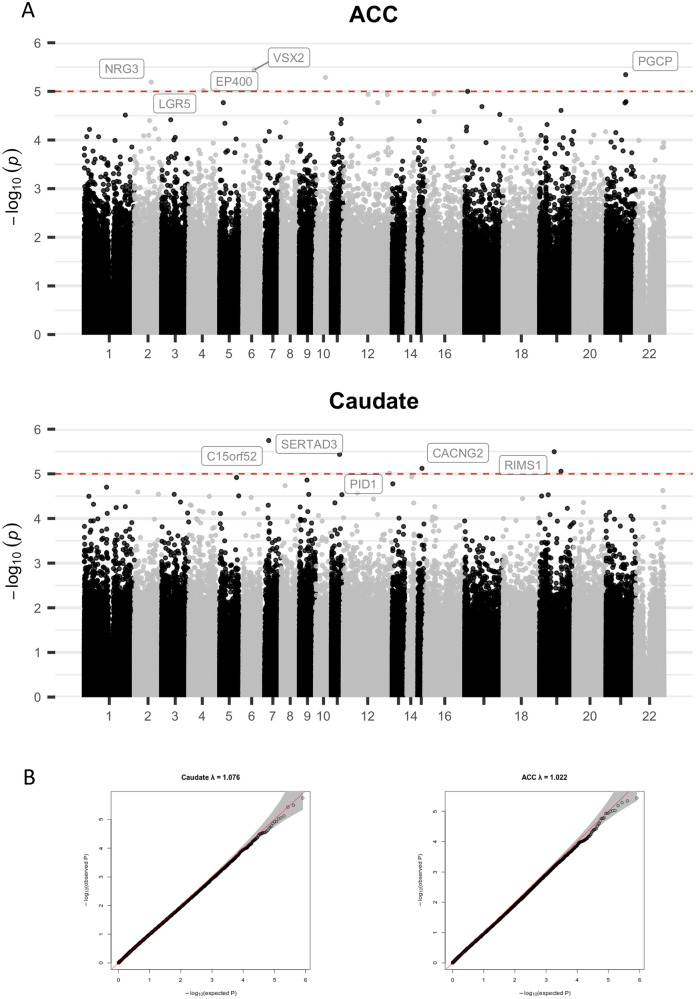
Results of a methylation-wide association study with ADHD. **A** Probe-level results annotated to the closest gene. The red line indicates nominal significant (at *p* < 1e–05). **B** qq plots, with lambda indicating no genomic inflation/deflation. ACC anterior cingulate cortex.

Clinical and demographic details of the donors are given in Table 1. In a model that showed no genomic inflation or deflation (all lambda < 1.08), no differentially methylated probes (DMPs) reached epigenome-wide significance at FDR q < 0.05 in either the ACC or the caudate. However, 38,797 probes were nominally significant (at *p* < 0.05) in the caudate and 35,622 probes in the ACC - Fig. 1 and Supplementary File 1.

**Table 1:** Demographic and clinical details of the 58 donors.

		ADHD	Unaffected	Test of difference
Total N		25	33	
Age at death (years)	Mean (SD)	21 (8.4)	23 (8.1)	t = 0.9, *p* = 0.37
Sex	Male	22	24	Exact *p* = 0.14
	Female	3	9	
Race/ethnicity	White, non-Hispanic	19	13	X(3)2 = 7.7, *p* = 0.006
	Other	6	20	
Comorbidities	Depression	6	0	Exact *p* = 0.004
	Adjustment disorder	2	0	
	Bipolar affective disorder, not otherwise specified	1	0	
	Dysthymia	0	1	
	Autistic spectrum disorder	1	0	
Substance use disorders	Yes	12	0	Exact *p* < 0.001
	No	13	33	
Manner of death	Accident	9	8	X(3)2 = 8.2, *p* = 0.04
	Homicide	1	9	
	Suicide	8	4	
	Natural	7	12	
Post-mortem interval (hours)	Mean (SD)	27 (16)	20.4 (10.5)	t = 1.99, *p* = 0.05

### Differentially methylated regions

Using mCSEA we found differentially methylated regions (DMRs) significant at FDR q < 0.05 for the caudate (in 143 promoter regions, 222 in gene bodies, and 146 in CpG islands) and the ACC (221 DMRs in promoters, 381 in genes, and 340 in CpG islands) - Supplemental File 2. Three examples of these DMRs are shown in Supplemental Fig. 1. Genes implicated by DMRs in the caudate overlapped significantly with those for the ACC, with 104 intersecting genes (p < 3.77e^–62^, Jaccard index = 0.094).

**Fig. 2:**
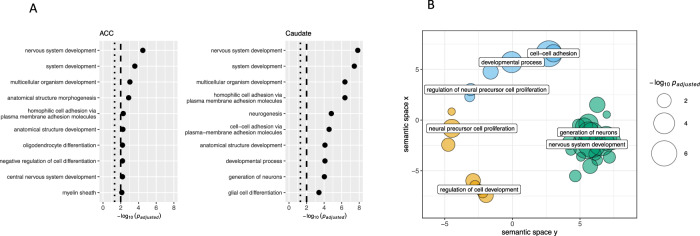
Gene set analyses results. **A** Top 10 biological processes that were most enriched by genes implicated through differential methylation. **B** A semantic space analysis of all significant biological processes (FDR *q* < 0.05), further highlighting the enrichment of genes pertinent to neurodevelopment.

In gene set analyses that included the leading methylation probes (at FDR q < 0.05), neurodevelopmental processes emerged as strongly enriched. This enrichment was noted for both brain regions, and ranged from gene pathways involved in neurogenesis, to the development of oligodendrocytes and glial cells. The top ten most significantly enriched gene sets in each brain region are shown in Fig. 2A, and Supplementary File 3 gives the full results. Processes of cell-cell adhesion were also enriched in both regions. Semantic analysis (REVIGO) of the Biological Process gene sets significantly enriched in the caudate also confirmed the over-representation of genes related to the nervous system development and neural precursor cell proliferation (Fig. 2B).

### Overlap between brain methylome and brain transcriptome in ADHD

We next used *mCSEAIntegrate* to determine if genes implicated by differential methylation of promoters, gene bodies or CGIs had correlated change in gene expression in the same brain regions in the same individuals. Correlations were generally modest and were higher for gene expression and methylation change in promoter regions than methylation in gene bodies or CGIs, both for the ACC (F(2, 33) = 7.84, *p* < 0.0004; promoter r = 0.17 [SD 0.16] > CGI, r = 0.13 [0.09] > gene bodies, r = 0.11 [0.08], all p < 0.05) and the caudate (F(2, 298) = 4.31, *p* < 0.01; promoter, r = 0.15 [0.11] > CGI, r = 0.14 [0.12] = gene bodies, r = 0.11 [0.08], *p* < 0.05).

Among genes showing correlated change (at FDR q < 0.05) in the methylome and transcriptome in both brain regions was *AURKC* (see Supplementary Fig. 2)—a gene involved in spindle formation during mitosis. In the ACC only, we found significant correlation for *TBX5*, which encodes transcription factors involved in the regulation of developmental processes, *VWDE*, involved in anatomic structure development, as well as *RGMA*, a gene involved in neurite outgrowth, cortical neuron branching, and the formation of mature synapses.

### Associations between brain methylome and GWAS for ADHD and other psychiatric disorders

**Fig. 3:**
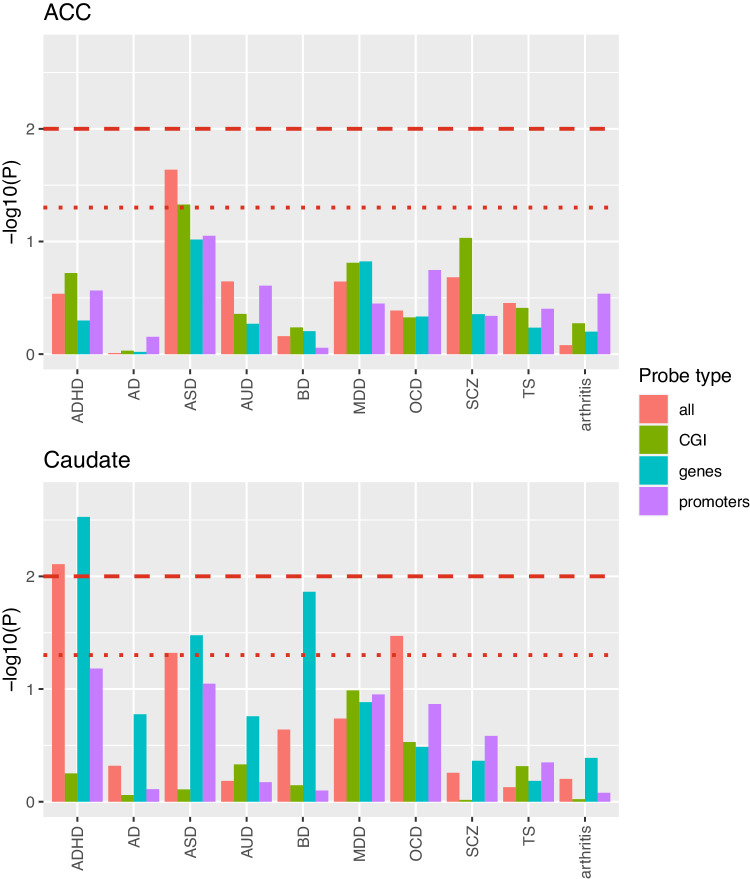
The results of MAGMA analyses determining if genes implicated by DMRs were associated with genes for ADHD implicated through GWAS. The bold dashed red line indicates *p* = 0.01 and the dotted red line indicates *p* < 0.05. ASD autistic spectrum disorder, AUD alcohol use disorders, AD Alzheimer’s, BD bipolar affective disorder, MDD major depressive disorder, OCD obsessive compulsive disorder, SCZ schizophrenia, TS Tourette’s syndrome.

We next examined associations between differentially methylated genes and genes implicated by prior GWAS. We found significant associations between genes implicated in ADHD by mCSEA and genes implicated in ADHD through GWAS for the caudate, but not for the ACC- Fig. 3. There were also significant associations between genes implicated by GWAS for autism spectrum disorder and the DMRs for the caudate and for the ACC (at *p* < 0.05 only). More modest nominally significant (*p* < 0.05) associations were noted with bipolar disorder and obsessive compulsive disorder for the caudate genes.

## Discussion

In an effort to map methylation differences in the brain tied to ADHD, we found several differentially methylated regions (DMRs) that predominantly implicated neurodevelopmental gene pathways. We demonstrate a significant association between differentially methylated genes in the caudate and genes implicated by GWAS not only in ADHD but also in autistic spectrum, obsessive compulsive and bipolar affective disorders through GWAS.

The genes implicated by differential methylation pertained almost exclusively to neurodevelopmental processes, encompassing neurogenesis, and oligodendrocyte and glial cell differentiation. This finding is consistent with prior studies of the ADHD peripheral methylome which also point to neurodevelopmental gene pathways, particularly when the methylome is characterized at birth using cord blood [6, 7]. Our methylation findings also echo our earlier post-mortem finding that altered gene expression in ADHD was most prominent among genes known to be preferentially expressed during early life, particularly in the caudate [51].

We found a modest correlation between gene expression (using TWAS) and a gene-level metric of methylation. Some of the genes showing the most correlated changes in methylation level and expression are involved in neurodevelopmental processes, such as neurite outgrowth (*RCMA*) and anatomic development (*VWDF*). Previous post-mortem aligning methylation and gene expression patterns are limited, and generally also report modest correlation at the level of individual genes, with more robust links at the level of gene networks [51, 52].

We found that the genes implicated by differential methylation of gene bodies in the caudate aligned with genes implicated by GWAS in ADHD [1]. This finding is perhaps unsurprising as twin studies demonstrate that methylation is to some degree under genetic control [53–55], and thus common genetic variation conferring risk for ADHD would be expected to have a central methylomic reflection. It is interesting that the methylome of the caudate aligns with common variant genetic risk for the disorder, given that the pathophysiological events in the caudate has been linked to the onset of childhood ADHD in several theoretical models [56–60]. By contrast, the methylome of the ACC was not strongly associated with common variant risk for ADHD, possibly as the ACC may be more tied to the adult trajectories of ADHD, which only partially overlap with the risk genes for ADHD onset [60].

We note six limitations. First, while the postmortem brain specimens were acquired from multiple study sites, we tried to minimize heterogeneity effects by using a common pipeline for tissue preparation, methylation data acquisition and analysis. Second, we did not have fresh peripheral blood or saliva on the same subjects for methylomic study and so were unable to assess the degree of concordance between ADHD related change in the central and peripheral methylomes. Third, we assessed methylation in bulk tissue homogenates, and so our EWAS model incorporates estimated proportion of neurons as a covariate. Fourth, methylation is only one of the epigenetic mechanisms that impacts gene expression and we did not consider histone modification, acetylation or hydroxymethylation [61]. Multi-omic profiling of these epigenetic markers, ideally tied to changes in gene expression at the level of single cells is the next step for this work. In a similar vein, we note that the EPIC arrays have limited coverage of the methylome, assaying around ~900k CpGs. Superior cover is provided by approaches such as whole-genomic bisulphite sequencing (capturing around 28 million CpGs) or Methylation Capture Sequencing (capturing ~3.7 million CpGs), but these methods are much more expensive and require more genomic DNA [62]. Fifth, our cohort was racially diverse, which while a strength, required us to control for population stratification effects through the use of genetic principal components reflecting ancestry. Finally, the sample size was modest, reflecting the complexity of obtaining postmortem tissue and was only available on older adolescents and young adults. Given the sample size we could not explore the possible effects of psychostimulant medications on the brain’s methylome. We also focused on just two brain regions, and while these were chosen on theoretical grounds, it is possible that ADHD related changes may be more prominent in other areas and at other developmental stages, such as earlier in childhood.

In conclusion, we report the first study of the postmortem cortico-striatal methylome in ADHD, finding that differential methylation pointed to genes involved in brain development.

### Supplementary information

Supplemental methods

Suplemental figure 1

Supplemental figure 2

Supplemental file 1

Supplemental file 2

Supplemental file 3

## Data Availability

Data are being deposited in NIMH Data Archive under Collection 3151, experiment 2443 (https://nda.nih.gov/edit_collection.html?id=3151).

## References

[1] Demontis D, Walters GB, Athanasiadis G, Walters R, Therrien K, Nielsen TT (2023). Genome-wide analyses of ADHD identify 27 risk loci, refine the genetic architecture and implicate several cognitive domains. Nat Genet.

[2] Harich B, van der Voet M, Klein M, Čížek P, Fenckova M, Schenck A (2020). From rare copy number variants to biological processes in ADHD. Am J Psychiatry.

[3] Nigg JT (2023). Considerations toward an epigenetic and common pathways theory of mental disorder. J Psychopathol Clin Sci.

[4] Yao B, Christian KM, He C, Jin P, Ming GL, Song H (2016). Epigenetic mechanisms in neurogenesis. Nat Rev Neurosci.

[5] Spiers H, Hannon E, Schalkwyk LC, Smith R, Wong CC, O’Donovan MC (2015). Methylomic trajectories across human fetal brain development. Genome Res.

[6] Neumann A, Walton E, Alemany S, Cecil C, González JR, Jima DD (2020). Association between DNA methylation and ADHD symptoms from birth to school age: a prospective meta-analysis. Transl Psychiatry.

[7] Walton E, Pingault JB, Cecil CA, Gaunt TR, Relton CL, Mill J (2017). Epigenetic profiling of ADHD symptoms trajectories: a prospective, methylome-wide study. Mol Psychiatry.

[8] Meijer M, Klein M, Hannon E, van der Meer D, Hartman C, Oosterlaan J (2020). Genome-wide DNA methylation patterns in persistent attention-deficit/hyperactivity disorder and in association with impulsive and callous traits. Front Genet.

[9] Chen YC, Sudre G, Sharp W, Donovan F, Chandrasekharappa SC, Hansen N (2018). Neuroanatomic, epigenetic and genetic differences in monozygotic twins discordant for attention deficit hyperactivity disorder. Mol Psychiatry.

[10] Rovira P, Sánchez-Mora C, Pagerols M, Richarte V, Corrales M, Fadeuilhe C (2020). Epigenome-wide association study of attention-deficit/hyperactivity disorder in adults. Transl Psychiatry.

[11] van Dongen J, Zilhão NR, Sugden K, Hannon EJ, Mill J, Caspi A (2019). Epigenome-wide association study of attention-deficit/hyperactivity disorder symptoms in adults. Biol Psychiatry.

[12] Mooney MA, Ryabinin P, Wilmot B, Bhatt P, Mill J, Nigg JT (2020). Large epigenome-wide association study of childhood ADHD identifies peripheral DNA methylation associated with disease and polygenic risk burden. Transl Psychiatry.

[13] Wilmot B, Fry R, Smeester L, Musser ED, Mill J, Nigg JT (2016). Methylomic analysis of salivary DNA in childhood ADHD identifies altered DNA methylation in VIPR2. J Child Psychol Psychiatry.

[14] Amin V, Harris RA, Onuchic V, Jackson AR, Charnecki T, Paithankar S (2015). Epigenomic footprints across 111 reference epigenomes reveal tissue-specific epigenetic regulation of lincRNAs. Nat Commun.

[15] Nardone S, Sharan Sams D, Reuveni E, Getselter D, Oron O, Karpuj M (2014). DNA methylation analysis of the autistic brain reveals multiple dysregulated biological pathways. Transl Psychiatry.

[16] Numata S, Ye T, Herman M, Lipska BK (2014). DNA methylation changes in the postmortem dorsolateral prefrontal cortex of patients with schizophrenia. Front Genet.

[17] Wockner LF, Noble EP, Lawford BR, Young RM, Morris CP, Whitehall VLJ (2014). Genome-wide DNA methylation analysis of human brain tissue from schizophrenia patients. Transl Psychiatry.

[18] Hart H, Radua J, Nakao T, Mataix-Cols D, Rubia K (2013). Meta-analysis of functional magnetic resonance imaging studies of inhibition and attention in attention-deficit/hyperactivity disorderexploring task-specific, stimulant medication, and age effectsADHD functional MR imaging studies meta-analysis. JAMA Psychiatry.

[19] Hoogman M, Bralten J, Hibar DP, Mennes M, Zwiers MP, Schweren LSJ (2017). Subcortical brain volume differences in participants with attention deficit hyperactivity disorder in children and adults: a cross-sectional mega-analysis. Lancet Psychiatry.

[20] Hoogman M, Muetzel R, Guimaraes JP, Shumskaya E, Mennes M, Zwiers MP (2019). Brain imaging of the cortex in ADHD: a coordinated analysis of large-scale clinical and population-based samples. Am J Psychiatry.

[21] Hart H, Radua J, Nakao T, Mataix-Cols D, Rubia K (2013). Meta-analysis of functional magnetic resonance imaging studies of inhibition and attention in attention-deficit/hyperactivity disorder: exploring task-specific, stimulant medication, and age effects. JAMA Psychiatry.

[22] Volkow ND, Wang GJ, Newcorn J, Fowler JS, Telang F, Solanto MV (2007). Brain dopamine transporter levels in treatment and drug naïve adults with ADHD. Neuroimage.

[23] Volkow ND, Wang GJ, Kollins SH, Wigal TL, Newcorn JH, Telang F (2009). Evaluating dopamine reward pathway in ADHD: clinical implications. JAMA.

[24] Spencer TJ, Biederman J, Madras BK, Dougherty DD, Bonab AA, Livni E (2007). Further evidence of dopamine transporter dysregulation in ADHD: a controlled PET imaging study using altropane. Biol Psychiatry.

[25] Demontis D, Walters RK, Martin J, Mattheisen M, Als TD, Agerbo E (2019). Discovery of the first genome-wide significant risk loci for attention deficit/hyperactivity disorder. Nat Genet.

[26] Elia J, Glessner JT, Wang K, Takahashi N, Shtir CJ, Hadley D (2011). Genome-wide copy number variation study associates metabotropic glutamate receptor gene networks with attention deficit hyperactivity disorder. Nat Genet.

[27] Sudre G, Gildea DE, Shastri GG, Sharp W, Jung B, Xu Q (2023). Mapping the cortico-striatal transcriptome in attention deficit hyperactivity disorder. Mol Psychiatry.

[28] Mattheisen M, Grove J, Als TD, Martin J, Voloudakis G, Meier S (2022). Identification of shared and differentiating genetic architecture for autism spectrum disorder, attention-deficit hyperactivity disorder and case subgroups. Nat Genet.

[29] Wu Y, Cao H, Baranova A, Huang H, Li S, Cai L (2020). Multi-trait analysis for genome-wide association study of five psychiatric disorders. Transl psychiatry.

[30] Yang Z, Wu H, Lee PH, Tsetsos F, Davis LK, Yu D (2019). Cross-disorder GWAS meta-analysis for attention deficit/hyperactivity disorder, autism spectrum disorder, obsessive compulsive disorder, and Tourette Syndrome. bioRxiv.

[31] Lehne B, Drong AW, Loh M, Zhang W, Scott WR, Tan ST (2015). A coherent approach for analysis of the Illumina HumanMethylation450 BeadChip improves data quality and performance in epigenome-wide association studies. Genome Biol.

[32] Elliott HR, Tillin T, McArdle WL, Ho K, Duggirala A, Frayling TM (2014). Differences in smoking associated DNA methylation patterns in South Asians and Europeans. Clin Epigenetics.

[33] Mansell G, Gorrie-Stone TJ, Bao Y, Kumari M, Schalkwyk LS, Mill J (2019). Guidance for DNA methylation studies: statistical insights from the Illumina EPIC array. BMC genomics.

[34] Guintivano J, Aryee MJ, Kaminsky ZA (2013). A cell epigenotype specific model for the correction of brain cellular heterogeneity bias and its application to age, brain region and major depression. Epigenetics.

[35] Martorell-Marugán J, González-Rumayor V, Carmona-Sáez P (2019). mCSEA: detecting subtle differentially methylated regions. Bioinformatics.

[36] Snijders C, Maihofer AX, Ratanatharathorn A, Baker DG, Boks MP, Geuze E (2020). Longitudinal epigenome-wide association studies of three male military cohorts reveal multiple CpG sites associated with post-traumatic stress disorder. Clin Epigenetics.

[37] Martins-Ferreira R, Leal B, Chaves J, Li T, Ciudad L, Rangel R (2022). Epilepsy progression is associated with cumulative DNA methylation changes in inflammatory genes. Prog Neurobiol.

[38] Holland P, Wildhagen M, Istre M, Reiakvam OM, Dahl JA, Søraas A (2022). Cri du chat syndrome patients have DNA methylation changes in genes linked to symptoms of the disease. Clin Epigenetics.

[39] Zheng Y, Lunetta KL, Liu C, Katrinli S, Smith AK, Miller MW (2022). An evaluation of the genome-wide false positive rates of common methods for identifying differentially methylated regions using illumina methylation arrays. Epigenetics.

[40] Geeleher P, Hartnett L, Egan LJ, Golden A, Raja Ali RA, Seoighe C (2013). Gene-set analysis is severely biased when applied to genome-wide methylation data. Bioinformatics.

[41] Supek F, Bošnjak M, Škunca N, Šmuc T (2011). REVIGO summarizes and visualizes long lists of gene ontology terms. PLoS One.

[42] de Leeuw CA, Mooij JM, Heskes T, Posthuma D (2015). MAGMA: generalized gene-set analysis of GWAS data. PLoS Comput Biol.

[43] Auton A, Brooks LD, Durbin RM, Garrison EP, Kang HM, Korbel JO (2015). A global reference for human genetic variation. Nature.

[44] Grove J, Ripke S, Als TD, Mattheisen M, Walters RK, Won H (2019). Identification of common genetic risk variants for autism spectrum disorder. Nat Genet.

[45] Howard DM, Adams MJ, Clarke TK, Hafferty JD, Gibson J, Shirali M (2019). Genome-wide meta-analysis of depression identifies 102 independent variants and highlights the importance of the prefrontal brain regions. Nat Neurosci.

[46] Wray NR, Ripke S, Mattheisen M, Trzaskowski M, Byrne EM, Abdellaoui A (2018). Genome-wide association analyses identify 44 risk variants and refine the genetic architecture of major depression. Nat Genet.

[47] Stahl EA, Breen G, Forstner AJ, McQuillin A, Ripke S, Trubetskoy V (2019). Genome-wide association study identifies 30 loci associated with bipolar disorder. Nat Genet.

[48] Trubetskoy V, Pardiñas AF, Qi T, Panagiotaropoulou G, Awasthi S, Bigdeli TB (2022). Mapping genomic loci implicates genes and synaptic biology in schizophrenia. Nature.

[49] Yu D, Sul JH, Tsetsos F, Nawaz MS, Huang AY, Zelaya I (2019). Interrogating the genetic determinants of tourette’s syndrome and other Tic disorders through genome-wide association studies. Am J Psychiatry.

[50] (OCGAS) IOCDFGCI-GaOCGAS. Revealing the complex genetic architecture of obsessive-compulsive disorder using meta-analysis. Mol Psychiatry. 2018;23:1181–8. 10.1038/mp.2017.154 PMC6660151 28761083

[51] Chen C, Zhang C, Cheng L, Reilly JL, Bishop JR, Sweeney JA (2014). Correlation between DNA methylation and gene expression in the brains of patients with bipolar disorder and schizophrenia. Bipolar Disord.

[52] Lin D, Chen J, Duan K, Perrone-Bizzozero N, Sui J, Calhoun V (2021). Network modules linking expression and methylation in prefrontal cortex of schizophrenia. Epigenetics.

[53] van Dongen J, Ehli EA, Slieker RC, Bartels M, Weber ZM, Davies GE (2014). Epigenetic variation in monozygotic twins: a genome-wide analysis of DNA methylation in buccal cells. Genes.

[54] Grundberg E, Meduri E, Sandling JK, Hedman ÅK, Keildson S, Buil A (2013). Global analysis of DNA methylation variation in adipose tissue from twins reveals links to disease-associated variants in distal regulatory elements. Am J Hum Genet.

[55] Oliva M, Demanelis K, Lu Y, Chernoff M, Jasmine F, Ahsan H (2023). DNA methylation QTL mapping across diverse human tissues provides molecular links between genetic variation and complex traits. Nat Genet.

[56] Sudre G, Mangalmurti A, Shaw P (2018). Growing out of attention deficit hyperactivity disorder: insights from the ‘remitted’brain. Neurosci Biobehav Rev.

[57] Shaw P, Sudre G (2021). Adolescent attention-deficit/hyperactivity disorder: understanding teenage symptom trajectories. Biol Psychiatry.

[58] Schulz KP, Li X, Clerkin SM, Fan J, Berwid OG, Newcorn JH (2017). Prefrontal and parietal correlates of cognitive control related to the adult outcome of attention-deficit/hyperactivity disorder diagnosed in childhood. Cortex.

[59] Halperin JM, Schulz KP (2006). Revisiting the role of the prefrontal cortex in the pathophysiology of attention-deficit/hyperactivity disorder. Psychol Bull.

[60] Pingault JB, Viding E, Galera C, Greven C, Zheng Y, RP, Rijsdijk et al. Genetic and environmental influences on the developmental course of attention-deficit/hyperactivity disorder symptoms from childhood to adolescence. JAMA Psychiatry 2015; 10.1001/jamapsychiatry.2015.0469 T. 10.1001/jamapsychiatry.2015.0469 PMC6328013 25945901

[61] De Sa Nogueira D, Merienne K, Befort K (2019). Neuroepigenetics and addictive behaviors: where do we stand?. Neurosci Biobehav Rev.

[62] Shu C, Zhang X, Aouizerat BE, Xu K (2020). Comparison of methylation capture sequencing and Infinium MethylationEPIC array in peripheral blood mononuclear cells. Epigenetics Chromatin.

