# Supplementary material for: Cortico-striatal differences in the epigenome in attention-deficit/ hyperactivity disorder
Source: Transl Psychiatry. 2024 Apr 11;14:189. doi: 10.1038/s41398-024-02896-x (PMC11009227; doi:10.1038/s41398-024-02896-x)
Supplement: Supplementary file 1 — Supplemental methods [file 41398_2024_2896_MOESM1_ESM.docx]

Supplementary methods.

Diagnostic process.

The diagnosis of ADHD was conducted by teams at each study site, through DSM based, clinician interviews with the next of kin. For the NIMH HBCC and Maryland Brain Collection, postmortem interviews were performed with at least one family member of the deceased by a trained psychiatrist, social worker, or other health care professional using the Structured Clinical Interview for DSM-IV-clinician version (First et al. 1997) along with psychiatric record reviews using the Diagnostic Evaluation after Death (Salzman et al. 1983). Where available other pertinent records such as Medical Examiner’s Office or medical records were summarized into a case note, which was reviewed by two psychiatrists who arrived at a consensus for DSM diagnoses (Donati et al. 2008). The University of Pittsburgh Brain Tissue Donation Program similarly conducts a postmortem interview with the family of the deceased using a structured interview for the DSM 5. Interview data are reviewed by a team of psychiatrists and psychologists along with medical records and publicly available records to arrive at a DSM diagnosis (Department of Psychiatry 2020). Data are being submitted to NDA and are available from the authors upon reasonable request.

Determination of population PCs.

To determine population PCs, participants were genotyped on DNA extracted from brain tissue using the Illumina HumanOmniExpressExome-8v1–4 array with genome build GRCh38. Calls were made using GenomeStudio version 2.0.3 and exported to PLINK format using GenomeStudio’s PLINK Input Report Plug-in v2.1.4. SNPs were filtered to exclude those with < 95% call rate, then samples with less than 98% call rate were removed, followed by another pass to remove SNPs with < 98% call rate. Finally, SNPs with minor allele frequency > = 0.05 or at Hardy–Weinberg equilibrium >= 10^−4^ were removed, and only autosomal markers were kept. Fifty-nine of the initial 60 participants with RNA data were genotyped and all passed genotyping QC steps. The participant without genotype data was retained by imputing their PCs from those within the same self-ascribed race/ethnicity (white, non-Hispanic). In total we retained 646,902 SNPs were retained for further analysis.

The TWAS data.

The gene expression study was conducted on good quality RNA extracted from the same subjects and the same brain regions. RNA-seq was conducted used Illumina NovaSeq 6000, 2×150bp following Ribo-Zero GOLD treatment to remove mitochondrial RNA and cytoplasmic rRNA. In the original TWAS, we used the same analytic approach as the current study and considered: demographic/clinical features (age at death, gender, comorbidities, mode of death, clinical evidence level), genotypic (the first five population components - C1 through C5), and technical covariates (RNA-seq batch and brain bank of origin, post-mortem interval, and RINe) for inclusion in the model, retaining those significantly associated with either predictors or gene levels. In the correlation analyses we used expression levels that were residualized for RINe and batch.

Department of Psychiatry, University of Pittsburgh. 2020. 'A Unique Approach to Lifespan Diagnosis: The Psychological Autopsy,'. <https://psychiatry.pitt.edu/unique-approach-lifespan-diagnosis-psychological-autopsy>.

Donati, Robert J., Yogesh Dwivedi, Rosalinda C. Roberts, Robert R. Conley, Ghanshyam N. Pandey, and Mark M. Rasenick. 2008. 'Postmortem brain tissue of depressed suicides reveals increased Gs alpha localization in lipid raft domains where it is less likely to activate adenylyl cyclase', *The Journal of neuroscience : the official journal of the Society for Neuroscience*, 28: 3042-50.

First, Michael B, Robert L Spitzer, MWJB Gibbon, and Janet BW Williams. 1997. "Structured clinical interview for DSM-IV clinical version (SCID-I/CV)." In.: Washington DC: American Psychiatric Press.

Salzman, S, J Endicott, P Clayton, and G Winokur. 1983. 'Diagnostic evaluation after death (DEAD)', *National Institute of Mental Health, Rockville*.
