# Supplementary material for: Cortico-striatal differences in the epigenome in attention-deficit/ hyperactivity disorder
Source: Transl Psychiatry. 2024 Apr 11;14:189. doi: 10.1038/s41398-024-02896-x (PMC11009227; doi:10.1038/s41398-024-02896-x)
Supplement: Supplementary file 2 — Suplemental figure 1 [file 41398_2024_2896_MOESM2_ESM.pptx]

## Slide 1
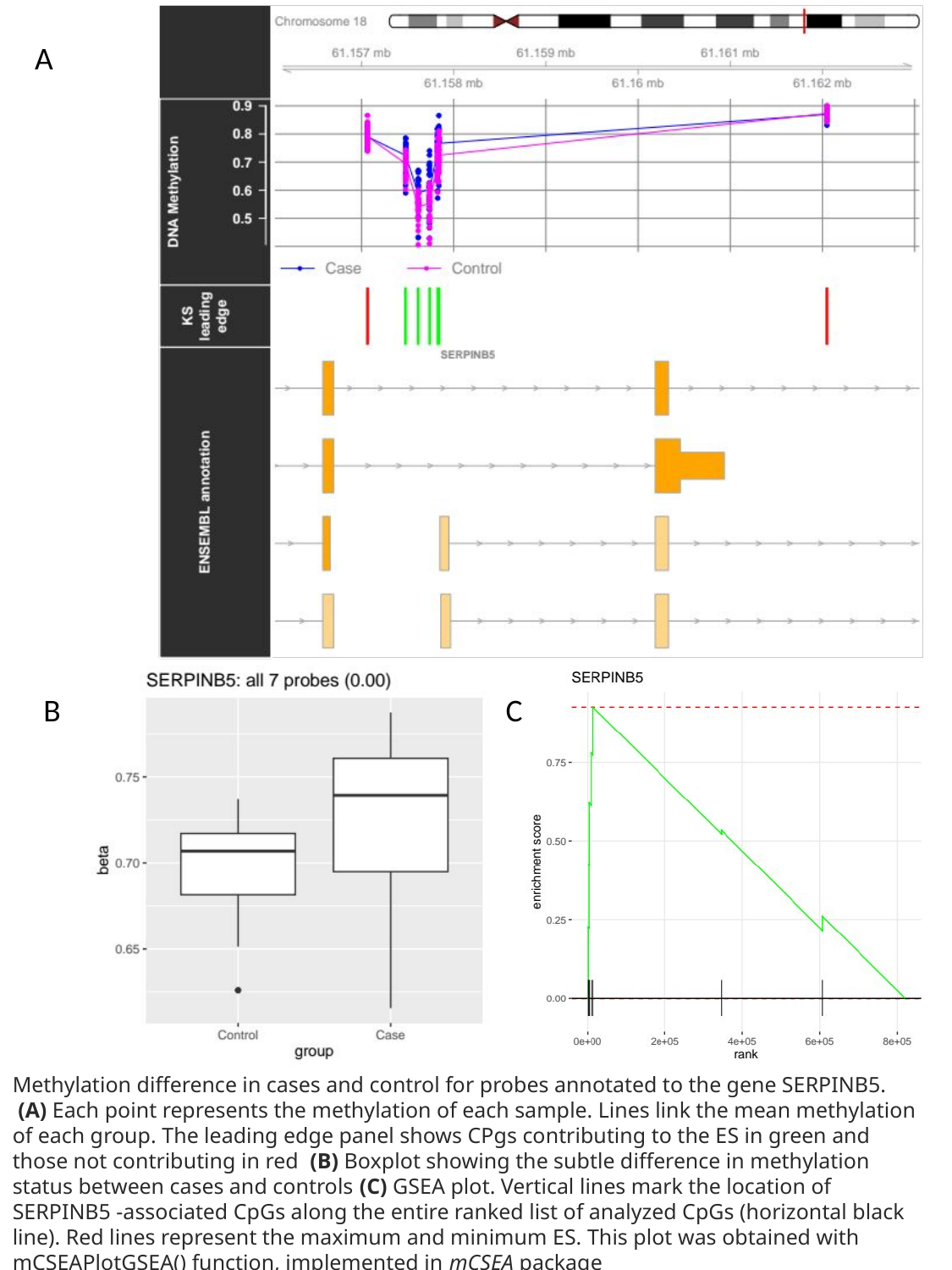

A
B
C
Methylation difference in cases and control for probes annotated to the gene SERPINB5.  (A) Each point represents the methylation of each sample. Lines link the mean methylation of each group. The leading edge panel shows CPgs contributing to the ES in green and those not contributing in red (B) Boxplot showing the subtle difference in methylation status between cases and controls (C) GSEA plot. Vertical lines mark the location of SERPINB5 -associated CpGs along the entire ranked list of analyzed CpGs (horizontal black line). Red lines represent the maximum and minimum ES. This plot was obtained with mCSEAPlotGSEA() function, implemented in mCSEA package

## Slide 2
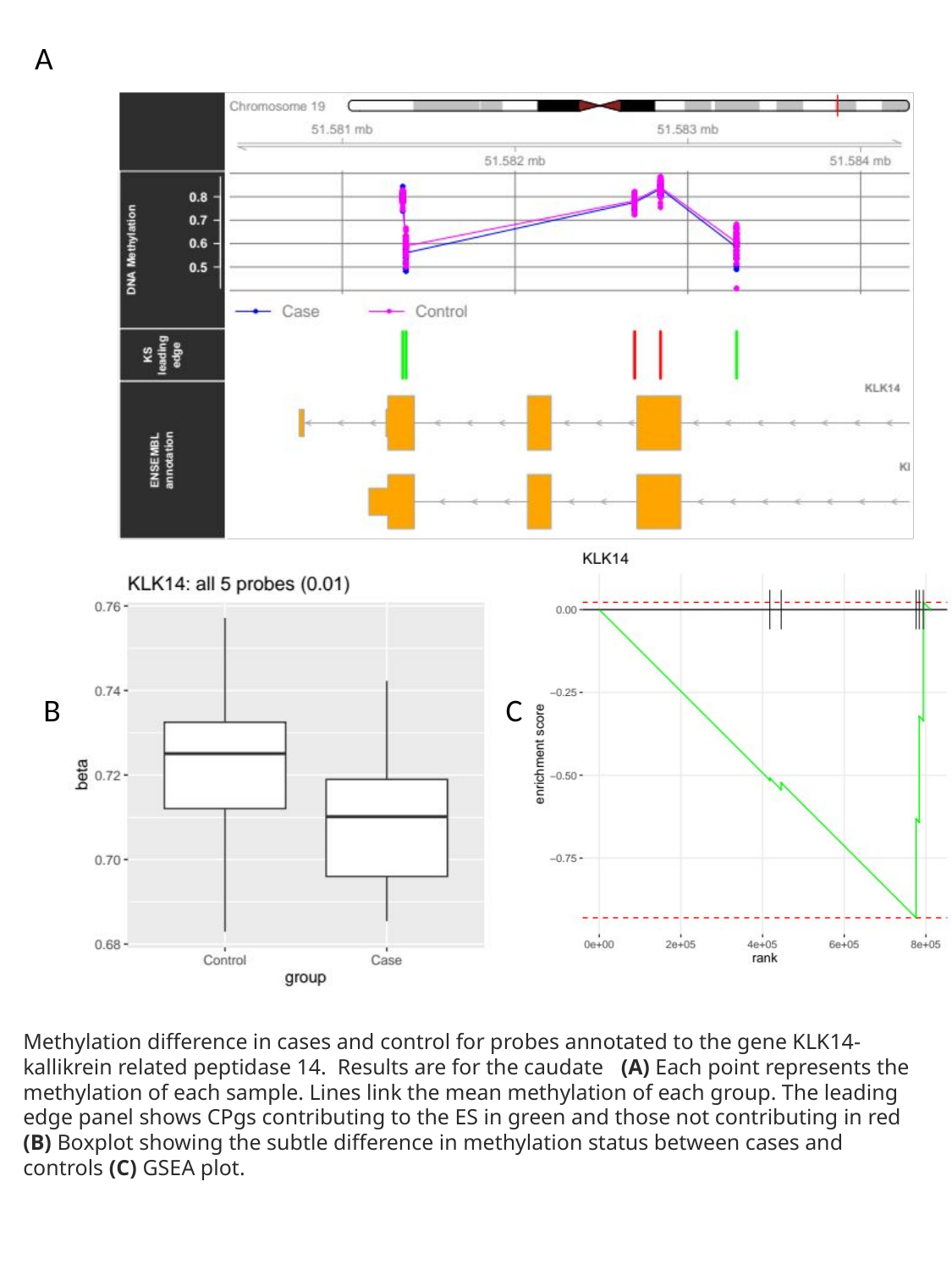

A
B
C
Methylation difference in cases and control for probes annotated to the gene KLK14- kallikrein related peptidase 14. Results are for the caudate  (A) Each point represents the methylation of each sample. Lines link the mean methylation of each group. The leading edge panel shows CPgs contributing to the ES in green and those not contributing in red (B) Boxplot showing the subtle difference in methylation status between cases and controls (C) GSEA plot.

## Slide 3
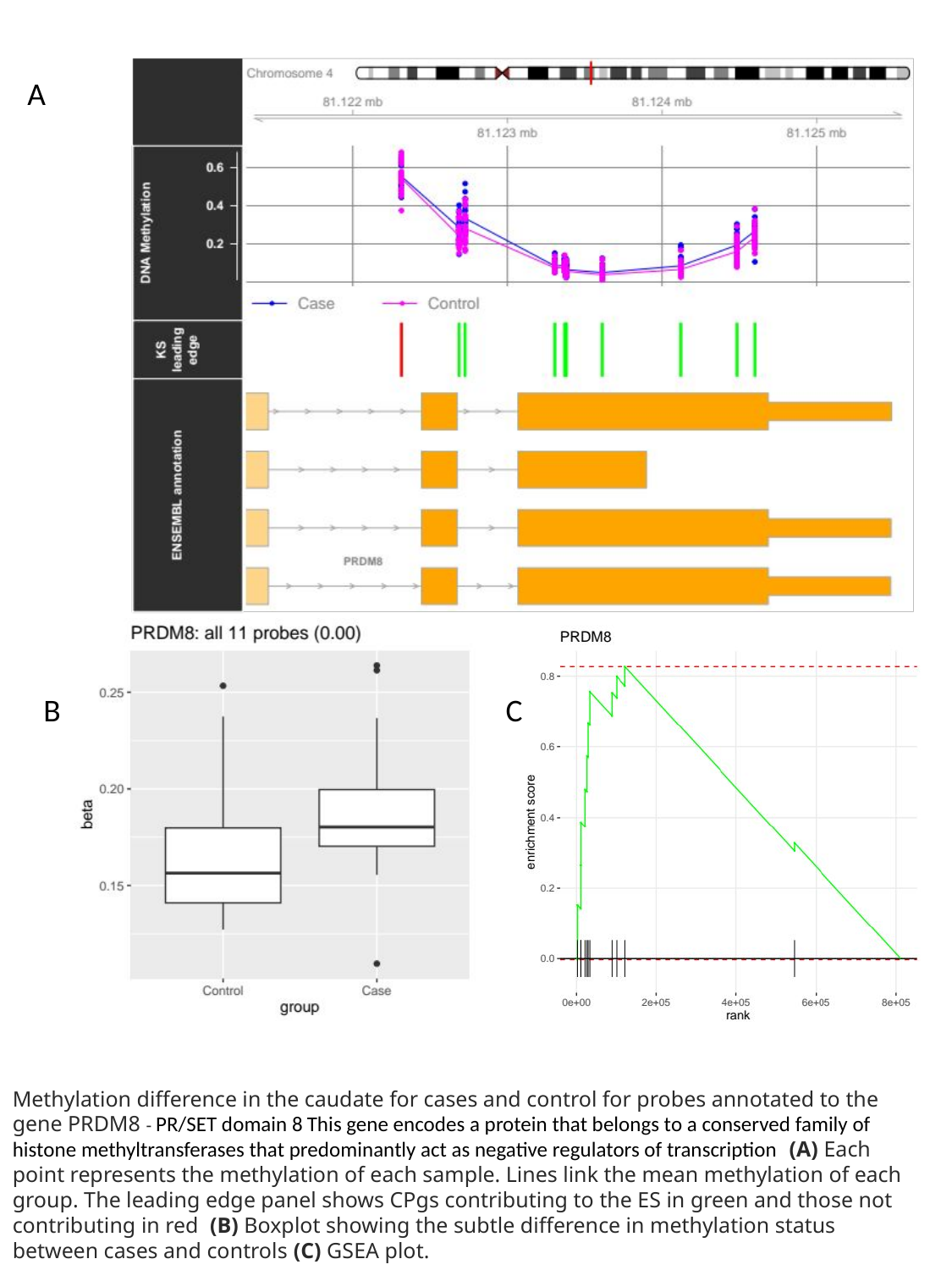

A
B
C
Methylation difference in the caudate for cases and control for probes annotated to the gene PRDM8 - PR/SET domain 8 This gene encodes a protein that belongs to a conserved family of histone methyltransferases that predominantly act as negative regulators of transcription  (A) Each point represents the methylation of each sample. Lines link the mean methylation of each group. The leading edge panel shows CPgs contributing to the ES in green and those not contributing in red (B) Boxplot showing the subtle difference in methylation status between cases and controls (C) GSEA plot.
