# Supplementary material for: Cortico-striatal differences in the epigenome in attention-deficit/ hyperactivity disorder
Source: Transl Psychiatry. 2024 Apr 11;14:189. doi: 10.1038/s41398-024-02896-x (PMC11009227; doi:10.1038/s41398-024-02896-x)
Supplement: Supplementary file 3 — Supplemental figure 2 [file 41398_2024_2896_MOESM3_ESM.pptx]

## Slide 1
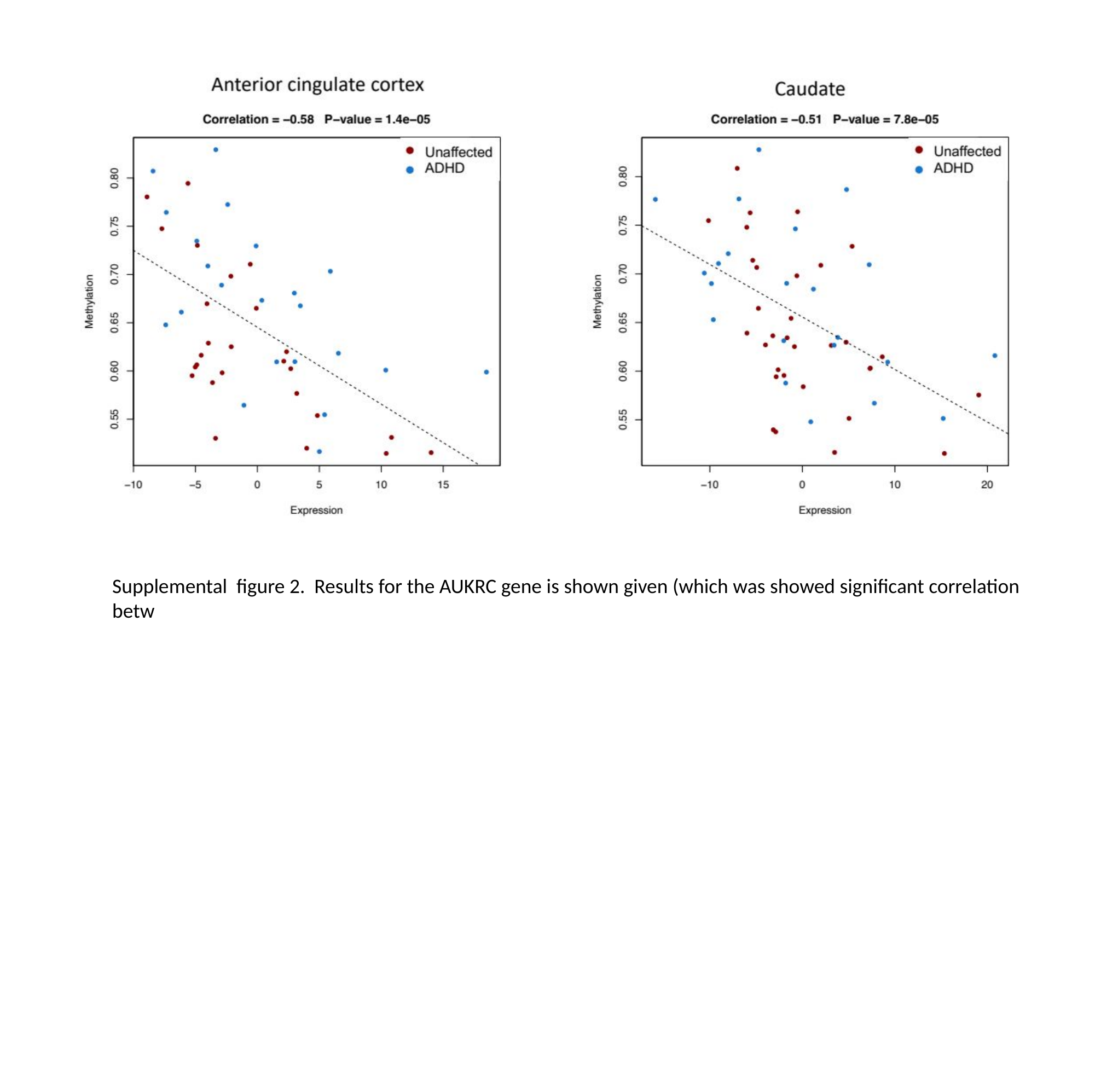

Supplemental figure 2. Results for the AUKRC gene is shown given (which was showed significant correlation betw
